# TRIP8b Is Required for Maximal Expression of HCN1 in the Mouse Retina

**DOI:** 10.1371/journal.pone.0085850

**Published:** 2014-01-07

**Authors:** Yuan Pan, Sajag Bhattarai, Modestos Modestou, Arlene V. Drack, Dane M. Chetkovich, Sheila A. Baker

**Affiliations:** 1 Department of Biochemistry, Carver College of Medicine, University of Iowa, Iowa City, Iowa, United States of America; 2 Department of Ophthalmology and Visual Sciences, Carver College of Medicine, University of Iowa, Iowa City, Iowa, United States of America; 3 The Ken & Ruth Davee Department of Neurology and Clinical Neurosciences and Department of Physiology, Feinberg School of Medicine, Northwestern University, Chicago, Illinois, United States of America; The University of Melbourne, Australia

## Abstract

Hyperpolarization-activated cyclic nucleotide-gated (HCN) channels are cation-selective channels present in retina, brain and heart. The activity of HCN channels contributes to signal integration, cell excitability and pacemaker activity. HCN1 channels expressed in photoreceptors participate in keeping light responses transient and are required for normal mesopic vision. The subcellular localization of HCN1 varies among cell types. In photoreceptors HCN1 is concentrated in the inner segments while in other retinal neurons, HCN1 is evenly distributed though the cell. This is in contrast to hippocampal neurons where HCN1 is concentrated in a subset of dendrites. A key regulator of HCN1 trafficking and activity is tetratricopeptide repeat-containing Rab8b interacting protein (TRIP8b). Multiple splice isoforms of TRIP8b are expressed throughout the brain and can differentially regulate the surface expression and activity of HCN1. The purpose of the present study was to determine which isoforms of TRIP8b are expressed in the retina and to test if loss of TRIP8b alters HCN1 expression or trafficking. We found that TRIP8b colocalizes with HCN1 in multiple retina neurons and all major splice isoforms of TRIP8b are expressed in the retina. Photoreceptors express three different isoforms. In TRIP8b knockout mice, the ability of HCN1 to traffic to the surface of retinal neurons is unaffected. However, there is a large decrease in the total amount of HCN1. We conclude that TRIP8b in the retina is needed to achieve maximal expression of HCN1.

## Introduction

Hyperpolarization-activated current (I_h_) was discovered in photoreceptors where absorption of light triggers a signal transduction cascade that leads to closure of cyclic nucleotide gated channels which in turn causes the cell to hyperpolarize. The subsequent opening of I_h_, an inward positive current helps to rapidly reset the membrane potential [1]–[6]. I_h_ is carried by hyperpolarization-activated and cyclic nucleotide-gated (HCN) channels that are expressed throughout the nervous system and the heart. HCN channels can serve as pacemakers, maintain resting membrane potential, shape synaptic output or modulate integration of dendritic signaling [7], [8]. There are four members of the HCN family (HCN1-4), all of which are found in the retina although with distinct expression profiles [9], [10].

Rod photoreceptors express HCN1 which is concentrated in the inner segments and to a lesser degree in the plasma membrane surrounding the nuclei (cell soma) and in the presynaptic terminals; HCN1 is excluded from the photosensitive outer segment compartment [10]–[13]. Cones express HCN1 in a similar pattern but also contain HCN3 at the synapse [10]. HCN1 is also expressed in multiple inner retina neurons, although seemingly less abundantly, and is found in multiple cellular compartments (dendrites, soma, axons and presynaptic terminals) [10]–[20]. In other neuronal populations HCN1 can be distributed throughout the cell or restricted to particular subcellular domains. For example, HCN1 is found in stereocilia and afferent dendrites of cochlear hair cells [21], [22] and in the soma and nodes of Ranvier in dorsal root ganglia [23]. But in hippocampal CA1 and layer V neocortical pyramidal neurons, HCN1 is concentrated in distal dendrites [24]. Since changes in HCN1 abundance or subcellular localization are associated with learning and memory, epilepsy, and pain [25], [26], it is important to understand how the trafficking of HCN1 is regulated in various cell types. The best understood regulator of HCN1 subcellular localization is tetratricopeptide repeat-containing Rab8b-interacting protein (TRIP8b).

TRIP8b is a cytoplasmic protein that binds to the C-terminus of HCN channel subunits via two contact sites [24], [27], [28]. TRIP8b interactions with the cyclic nucleotide binding domain of HCN allows it to modulate the gating and surface expression of HCN channels, while TRIP8b’s interaction with the last three amino acids of HCN C-terminus is important for proper trafficking of the channel [28]–[30]. Multiple splice isoforms of TRIP8b are expressed in the brain and can have opposing influences on the localization of HCN1 [26], [29]. In the hippocampus, TRIP8b is essential for maintaining the expression level, surface availability, and the concentration of HCN1 channels in distal dendrites of pyramidal neurons [26]. Furthermore, TRIP8b can inhibit the axonal distribution of HCN1 in the medial perforant path [31]. However, trafficking of HCN1 to presynaptic terminals in the cortex is independent of TRIP8b [32] so the role of TRIP8b can vary depending on neuronal population and subcellular localization. It is not known if TRIP8b regulates the trafficking of HCN1 channels in retinal neurons.

In this study we found that all major splice isoforms of TRIP8b are expressed in the retina and TRIP8b co-localizes with HCN1 in photoreceptors and inner retina neurons. In the absence of HCN1, TRIP8b is expressed at normal levels in the retina, but it is not fully recruited to membranes. Conversely, in the absence of all TRIP8b isoforms, HCN1 levels are reduced. Despite this, the trafficking of HCN1 to the surface of retinal neurons was maintained, and visual function as measured with ERG was normal in TRIP8b knockout animals. We conclude that trafficking of HCN1 channels in the retina can take place independent of TRIP8b but this accessory subunit is necessary to maintain maximal expression of the channel.

## Materials and Methods

### Animals

C57BL/6J pigmented wild type mice were purchased from the Jackson Laboratory as were the HCN1^−/−^ mice originally described by Nolan and colleagues [33]. The TRIP8b^−/−^ and TRIP8b 1b/2^−/−^ lines were maintained as previously described [26], [29], [31]. All three knockout lines are congenic on the C57BL/6 strain background. Mice were maintained on a standard 12/12 hour light/dark cycle; food and water were provided *ad libitum*. This study was carried out in strict accordance with the recommendations in the Guide for the Care and Use of Laboratory Animals of the National Institutes of Health. The experiments were approved by the Institutional Animal Care and Use Committee at the University of Iowa, and adhered to the ARVO guidelines for animal use in vision research. Prior to tissue collection, mice were humanely euthanized by CO_2_ asphyxiation followed by cervical dislocation and all efforts were made to minimize suffering.

### Immunohistochemistry

Individual mouse eyes were enucleated and eyecups were prepared by removing the cornea, iris and lens. Eyecups were fixed in 4% paraformaldehyde prepared in PBS at room temperature for 15 min, cryoprotected in 30% sucrose at 4°C O/N then frozen in O.C.T (Tissue-Tek) before collecting 10 µm cryosections. For immunostaining, the sections were permeablized in PBS containing 0.5% Triton X-100 for 10 min, followed by incubation in 10% goat serum to block nonspecific labeling. Eyecup sections were incubated with primary antibodies at 4°C O/N, washed and incubated with secondary antibodies conjugated to either Alexa-488 or Alexa-568, and Hoechst 33342 (Life Technologies) to label nuclei. The primary antibodies used were: rabbit α-HCN1 raised against a C-terminal epitope, NTNLTKEVRPLSAS, (developed at GenScript, for validation of the antibody see Figure S1), mouse α-HCN1 (NeuroMab, 1∶500), rabbit α-HCN1 (Alomone, 1∶500), rabbit α-TRIP8b-constant (1∶1000) [34], mouse α-TRIP8b-exon 4 (NeuroMab, 1∶1000). HCN1^−/−^ or TRIP8b^−/−^ retina were stained in parallel with wild type retina sections to ensure specificity of antibody labeling. Images were collected using a Zeiss 710 confocal microscope (Central Microscopy Research Facility, Univ. Iowa). Manipulation of images was limited to adjusting the brightness and contrast levels using Zen Light 2009 (Carl Zeiss) or Photoshop (Adobe). Experiments were replicated with a minimum of 3 individual mice.

### Laser Capture Micro-dissection

Mouse retinas were dissected as for immunohistochemistry, immediately frozen in O.C.T., sectioned and slides were stored at −80°C. All equipment was thoroughly cleaned with 70% ethanol followed by RNaseZap (Ambion) to minimize RNA degradation. Photoreceptor inner segments were collected with a 7.5 µm diameter laser beam on an Arcturus PIXCELL II Laser Capture Microscope (Central Microscopy Research Facility, Univ. Iowa). The selected regions were then absorbed by the Arcturus CapSure Macro LCM Caps (Applied Biosystems) and immediately processed for RNA extraction as described below. The experiments were replicated with a minimum of 4 individual mice.

### RT-PCR

RNA from one mouse retina was extracted with the RNeasy Mini kit (Qiagen), M-MLV reverse transcriptase (Life Technologies) with either random hexamers or gene-specific primers was used for cDNA synthesis, followed by standard PCR reactions to amplify specific products. After separation on agarose gels, all amplification fragments were extracted and verified by sequencing (Univ. Iowa DNA facility). The experiment was replicated 3 times. Primers used for RT-PCR: TRIP8b-exon 1a-forward, 5′gagcagaatgtaccagggacacat; TRIP8b-exon 1b-forward, 5′ggaaggactcacattccatctctac; TRIP8b-exon 5-reverse, 5′tggatgtcactggctttgcaatggc.

### Fractionation of Cytosolic and Membrane Proteins

Freshly isolated mouse retina was homogenized in hypotonic buffer (50 mM Tris-HCl, 10 mM NaCl, 0.32 M sucrose, 5 mM EDTA, 2.5 mM EGTA, pH7.4) supplemented with protease inhibitor cocktail (Complete, mini, Roche), followed by centrifugation at 1,000×g for 10 min at 4°C to pellet unbroken cells and nuclei. The supernatant was centrifuged at 240,000×g for 30 min at 4°C. The supernatant, containing cytosolic proteins, was collected. The pellet, containing membrane and cytoskeletal proteins, was resuspended in hypotonic buffer plus detergent (1.5% Triton X-100 and 0.75% DOC). After centrifugation at 240,000×g for 30 min at 4°C, the supernatant, containing solubilized membrane proteins, was collected. The experiment was replicated 3 times.

### Immunoprecipitation

Twenty retinas from either wild type or HCN1^−/−^ mice were homogenized in 20 mM HEPES, pH 7.4, 150 mM NaCl, 1 mM EDTA, 1 mM EGTA, and 1 mM PMSF containing complete protease inhibitor cocktail. The homogenate was centrifuged at 800×g for 10 min at 4°C to remove nuclei and the post-nuclear supernatant was centrifuged at 100,000×g for 1 h at 4°C to pellet the retinal membranes. The membrane fraction was solubilized in homogenization buffer supplemented with 1% DDM (n-Dodecyl-beta-D-Maltoside) for 1 h at 4°C on a rotator and subsequently centrifuged at 100,000×g for 1 h at 4°C to remove residual unsolubilized material. 250 µg of solubilized membrane extract was incubated with rabbit anti-HCN1 antibody for 2 h at 4°C on a rotator. After addition of 1.5 mg Dynabeads Protein G (Life Technologies), incubation was continued for an additional hour. Dynabeads were washed three times in homogenization buffer supplemented with 1% DDM to remove unbound proteins, transferred to a new tube, and bound proteins were eluted with NuPAGE LDS sample buffer (Life Technologies) lacking reducing agent. The experiment was replicated 3 times.

### Biotinylation Assay

Freshly isolated mouse retina (two for each experiment) was incubated with 1 mg/mL sulfo-NHS-SS-biotin (Thermo Scientific) for 10 min at 4°C to label only the surface proteins. The reaction was quenched (100 mM glycine, 25 mM Tris-HCl, pH7.4) for 15 min, and then washed in PBS three times; all procedures were carried out at 4°C. The biotinylated retinas were homogenized in lysis buffer (50 mM Tris-HCl, 10 mM NaCl, 0.32 M sucrose, 5 mM EDTA, 2.5 mM EGTA, 1.5% Triton X-100, 0.75% DOC, 0.1% SDS, pH7.4) supplemented with protease inhibitor cocktail (Complete, mini, Roche). Insoluble material was removed by centrifugation at 10,000×g for 10 min and the supernatant was incubated with 100 µl NeutrAvidin agarose resin (Thermo Scientific) with gentle mixing at 4°C O/N. Beads were washed with lysis buffer three times and proteins bound to the beads were eluted with a reducing NuPage LDS sample buffer (Life Technologies). The experiment was replicated with a minimum of 3 individual mice of each genotype.

### Western Blotting

Protein content in samples was measured using the BCA assay (Thermo Scientific). Proteins were fractionated on 10% Mini-PROTEAN TGX gels and transferred to PVDF membranes (Bio-Rad). Membranes were blocked in 5% milk and incubated with the following primary antibodies: rabbit α-HCN1 (3 µg/mL), rabbit α-TRIP8b (1∶10,000), mouse α-NKA (M7-PB-E9, Santa Cruz, 1∶1000), rabbit α-PDC (gift from Dr. Maxim Sokolov, 1∶3000) and secondary antibodies conjugated to HRP. Blots were incubated with SuperSignal West Femto Maximum Sensitivity Substrates (Thermo Scientific) and visualized with a CCD camera (ChemiDoc XR+, Bio-Rad). The software package Image Studio v3.1 (LI-COR Biosciences) was used for analysis of the images.

### Electroretinography

Full field ERG was obtained using the Espion V5 Diagnosys system (Diagnosys LLC, MA, USA). After overnight dark-adaptation, mice were anesthetized with an intraperitoneal injection of ketamine (87.5 mg/kg) and xylazine (2.5 mg/kg). Mice were 5–16 weeks of age, HCN1^−/−^ and TRIP8b^−/−^ animals were compared to wild type littermates obtained from breeding heterozygotes. ERGs were recorded simultaneously from the corneal surface of each eye after pupil dilation (1% tropicamide) using gold ring electrodes (Diagnosys) referenced to a needle electrode (Roland/LKC) placed on the back of the head. Another needle electrode placed in the tail served as the ground. A drop of methylcellulose (2.5%) was placed on the corneal surface to ensure electrical contact and to maintain corneal integrity. Body temperature was maintained at a constant temperature of 38°C using a regulated heating pad. All stimuli were presented in a ColorDome (Diagnosys) ganzfeld bowl and the mouse head and electrode positions were monitored on the camera attached to the system. Dim red light was used for room illumination until dark adapted testing was completed. A modified ISCEV protocol [35], [36] was used including a dark adapted dim flash of 0.01 cd.s/m^2^, maximal combined response (standard combined response or SCR) to bright flash of 3 cd.s/m^2^, light adapted bright flash of 3 cd.s/m^2^,and 5 Hz flicker stimuli at 3 cd.s/m^2^. An escalating intensity protocol was utilized with intensities starting at 0.0001 cd.s/m^2^ and increasing incrementally to 31.6 cd.s/m^2^. A dark-adapted increasing flicker frequency protocol was utilized in which the intensity of flash was held constant at 3.1 cd.s/m^2^ and the flash frequency increased from 0.5 Hz incrementally to 30 Hz. The a-wave was measured from the baseline to the trough of the first negative wave. The b-wave was measured from the trough of the a-wave to the peak of the first positive wave, or from the baseline to the peak of the first positive wave if no a-wave was present.

## Results

### Multiple TRIP8b Isoforms are Present in the Retina

**Figure 1:**
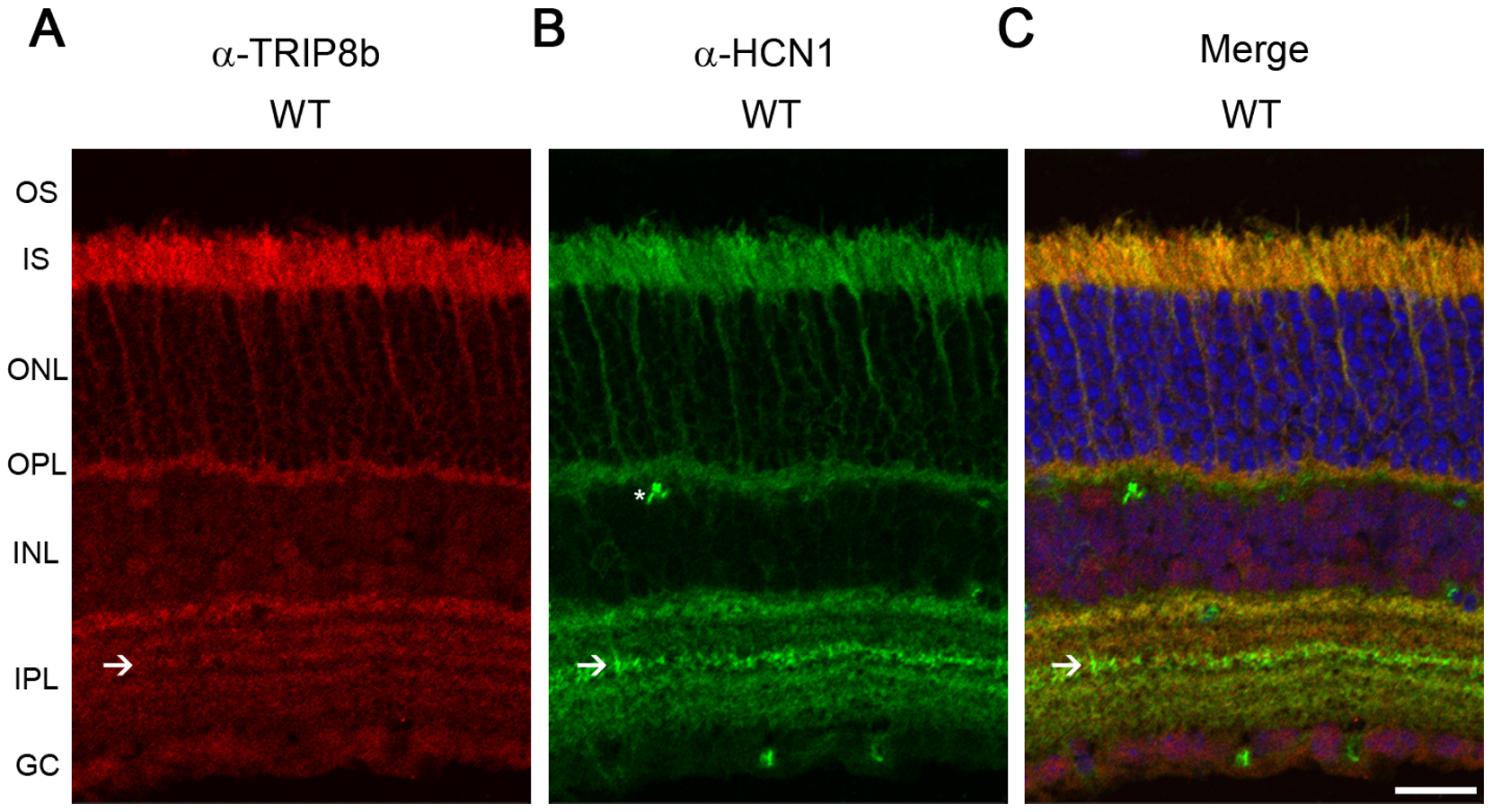
TRIP8b co-localizes with HCN1 in retina. Mouse retina immunostained with antibodies against *A)* TRIP8b (red), *B)* HCN1 (green), *C)* merged image demonstrating co-localization of these two proteins in the IS and OPL and partial co-localization in the IPL. Asterisks indicate non-specific labeling of blood vessels; arrows indicate IPL sublamina strongly labeled for HCN and containing TRIP8b. The nuclei are counterstained with Hoechst (blue). Abbreviations: OS, outer segment; IS, inner segment; ONL, outer nuclear layer; OPL, outer plexiform layer; INL, inner nuclear layer; IPL, inner plexiform layer; GC, ganglion cell layer. Scale bar is 20 µm.

TRIP8b is an accessory subunit of HCN1 channels that influences its trafficking and activity in various neurons [37], but its expression in the retina has not been investigated. Immunohistochemistry of the mouse retina revealed that TRIP8b is expressed in multiple regions throughout the retina; including photoreceptors with less intense labeling of the inner plexiform layer and ganglion cells (Figure 1A). In photoreceptors, TRIP8b is present in the inner segment (IS) and synapses of the outer plexiform layer (OPL) but excluded from outer segments – the same distribution as HCN1 (Figure 1B, C). In the inner plexiform layer (IPL), TRIP8b labeling could be distinguished in all sublamina, including the sublamina that is prominently stained by anti-HCN1 antibodies due to the expression of HCN1 in type 5 bipolar axon terminals [10], [12], [38]. This demonstrates that TRIP8b is expressed in cells containing HCN1, but not exclusively. This is reasonable given that HCN2 and HCN4 are also expressed in various sublamina of the IPL and can interact with TRIP8b [10], [38].

**Figure 2:**
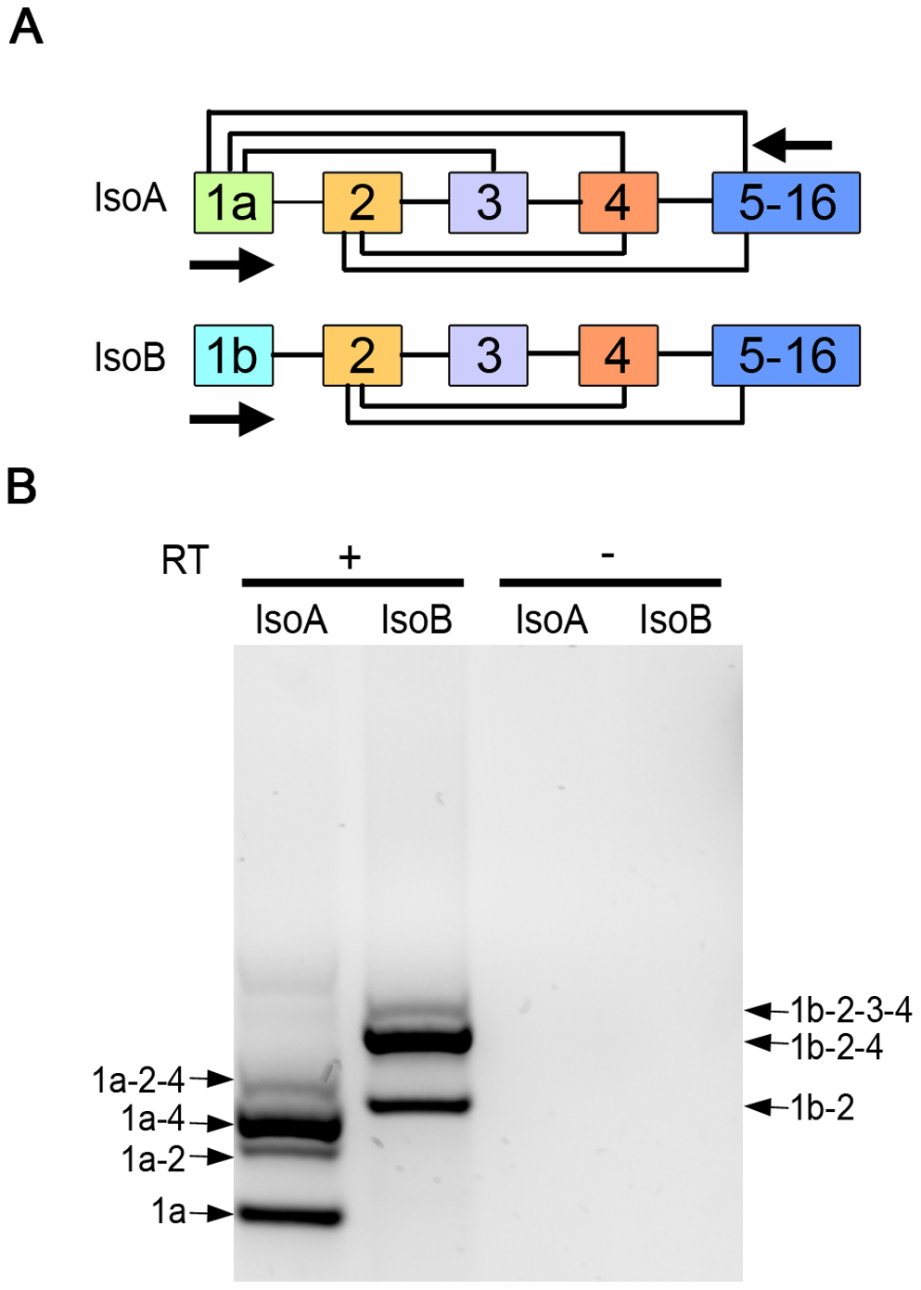
Multiple TRIP8b splice variants are expressed in retina. *A)* Illustration of the alternative splicing that can generate 6, exon 1a containing TRIP8b isoforms (IsoA) or 3, exon 1b TRIP8b isoforms (IsoB). Arrows indicate positions of primers used for RT-PCR. *B)* RT-PCR products obtained using RNA isolated from mouse retina and primer pairs to amplify TRIP8b IsoA (lane 1) or IsoB splice variants (lane 2). Reverse transcriptase was omitted as a negative control (lanes 3, 4).

TRIP8b is subject to alternative splicing generating 9 unique variants which can differentially regulate HCN1 [27], [39]. To determine which TRIP8b splice isoforms are expressed in the retina, we performed RT-PCR using RNA isolated from mouse retina and primer pairs designed to distinguish between the isoforms (Figure 2A). We found seven products in the retina (Figure 2B), that correspond to the major isoforms expressed throughout the brain. Each product was sequenced to verify that it was the expected TRIP8b splice variant.

**Figure 3:**
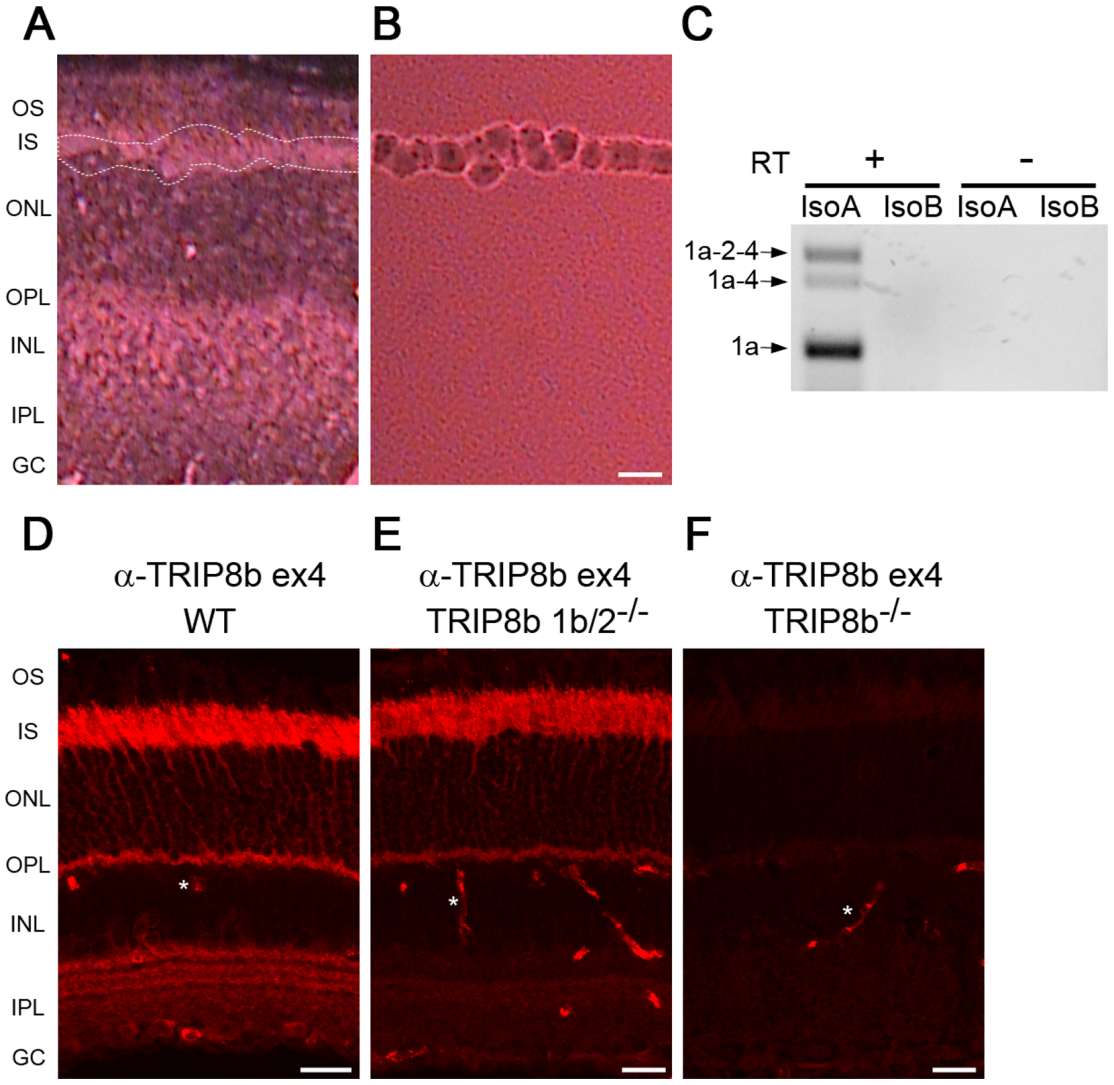
Multiple TRIP8b splice variants are expressed in photoreceptors. *A)* Mouse retina section before laser capture micro-dissection, dotted white lines indicate region subsequently collected and shown in *B*. *C)* RT-PCR products obtained using RNA isolated from tissue collected as in *B* with the isoform specific primers depicted in Figure 2A. Reverse transcriptase was omitted as a negative control. Immunostaining with an antibody recognizing exon 4 in TRIP8b labels photoreceptors of *D)* wild type, *E)* TRIP8b 1b/2^−/−^, but not *F)* TRIP8b^−/−^ mouse retina. Asterisks indicate non-specific labeling of blood vessels. Abbreviations as in Figure 1; Scale bars are 20 µm.

The retina is a multilayered tissue composed of five principle types of neurons and several types of glial cells. To determine which of the various TRIP8b isoforms are expressed in photoreceptors, we used laser capture micro-dissection of the mouse retina to collect the IS, the layer where the majority of photoreceptor transcripts are translated. The only other cell type possibly collected in this procedure would be a very small fraction of the Muller glial cells that span the retina from the ganglion cell layer to the outer limiting membrane between the IS and ONL layers. Imaging of the retina before and after micro-dissection was used to confirm the efficiency of IS isolation (Figure 3A, 3B). RT-PCR performed using RNA isolated from the IS revealed three isoforms: TRIP8b 1a (IsoA5), TRIP8b 1a-4 (IsoA4) and TRIP8b 1a-2-4 (IsoA2) (Figure 3C). This observation is consistent with the abundance of these transcripts in other neuronal populations, where TRIP8b 1a and 1a-4 together comprise ∼60% of all TRIP8b isoforms expressed in the brain with 1b containing isoforms concentrated in glial cells [29], [39].

The expression of exon 4 containing isoforms in photoreceptors can be confirmed by immunohistochemistry using an anti-TRIP8b antibody specific to an epitope in this exon. This antibody should label photoreceptors in wild type retina (isoforms 1a-4 and 1a-2-4) and in TRIP8b 1b/2 knockout retina (isoform 1a-4). As predicted, labeling of inner segments and the outer plexiform layer is similar to that seen with the anti-TRIP8b antibody that detects all isoforms (Figure 3D, E, compare to Figure 1A). Interestingly, the staining of the inner plexiform layer by the exon 4 specific antibody is lost in the TRIP8b 1b/2 knockout retina, indicating that this region is the source of the 1b-2-3-4 and 1b-2-4 isoforms detected by RT-PCR of the whole retina.

### Interaction of HCN1 and TRIP8b in the Retina

**Figure 4:**
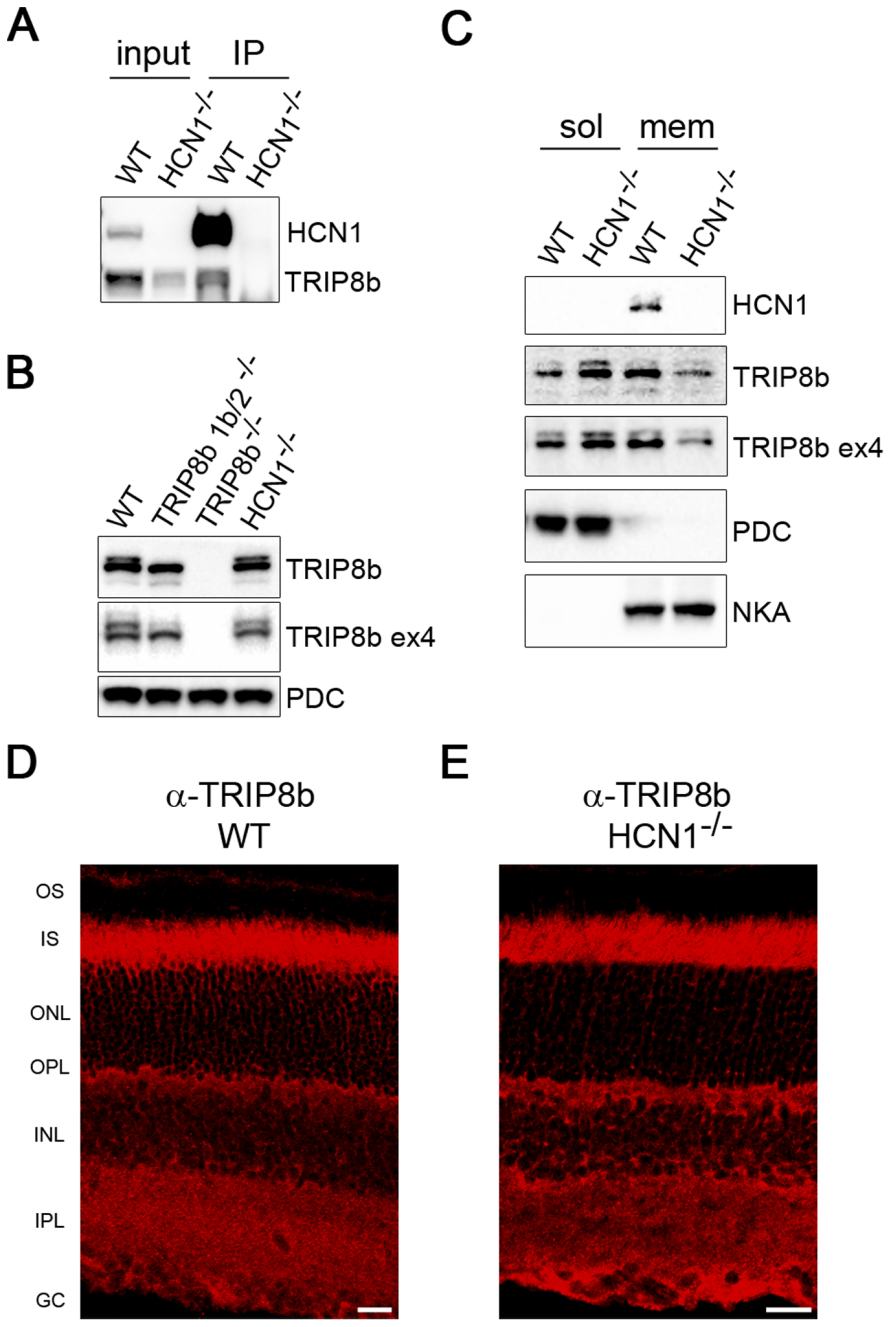
HCN1 is required to fully recruit TRIP8b to the membrane. *A)* Anti-HCN1 antibodies were used to co-immunoprecipitate TRIP8b from retinal membranes. Membranes prepared from HCN1^−/−^ retinas were used as the negative control. *B)* Western blot comparing the amount of TRIP8b present in total retina lysates from wild type, both TRIP8b knockout lines, and HCN1^−/−^ mice. Phosducin (PDC) is the loading control. *C)* Retina lysates from wild type and HCN1^−/−^ mice separated into cytosolic and membrane fractions probed with anti-TRIP8b and anti-HCN1 antibodies. PDC and sodium/potassium ATPase (NKA) are loading controls for each fraction. Immunostaining of TRIP8b in wild type (*D*) and HCN1^−/−^ retina (*E*) is indistinguishable. Abbreviations as in Figure 1; Scale bars are 20 µm.

HCN1 and TRIP8b interact in the retina as confirmed by immunoprecipitation from retinal membranes (Figure 4A). Membranes prepared from the retinas of HCN1^−/−^ were used for the negative control and we noted a reduction of TRIP8b in this preparation. When total retina extracts were probed with anti-TRIP8b antibodies (constant or exon-4 specific), the expression level of TRIP8b was found to be the same as that in wild type retinas (Figure 4B). Due to the similarity in molecular weight, the various TRIP8b isoforms are detected as just 2 or 3 bands on Western blots. To further explore the difference in TRIP8b levels observed in Figures 4A and 4B, we compared cytosolic and membrane fractions of the retina side by side; the efficiency of this separation was monitored by probing for a soluble protein, phosducin (PDC), and the membrane bound sodium potassium ATPase (NKA). There was a reduction in the amount of TRIP8b in the isolated membrane fraction and a corresponding increase in the cytosolic fraction from the HCN1^−/−^ mouse compared to the wild type (Figure 4C). This demonstrates that HCN1 is needed to fully recruit TRIP8b to retinal membranes. The remaining pool of membrane-associated TRIP8b likely reflects the recruitment of TRIP8b by HCN 2, 3, or 4. Note, as expected because the size of the photoreceptors does not provide the resolution needed to distinguish between plasma membrane and cytoplasm, there is no change in the pattern of TRIP8b immunostaining in the absence of HCN1 (Figure 4D, E).

**Figure 5:**
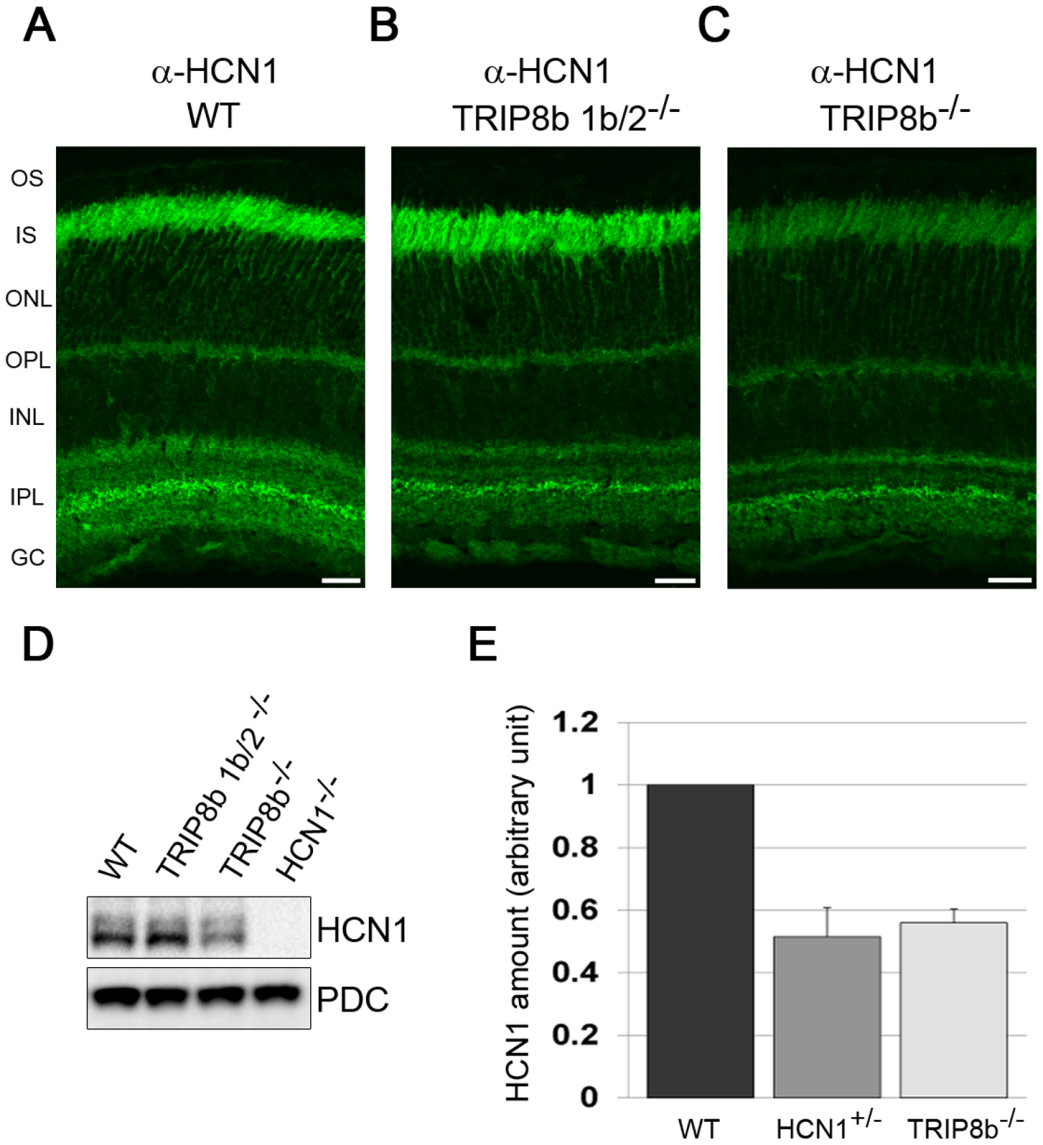
HCN1 protein levels are reduced in the absence of TRIP8b. Anti-HCN1 antibodies were used to immunostain retina from *A)* wild type *B)* TRIP8b 1b/2^−/−^, or *C)* TRIP8b^−/−^ mice. Abbreviations as in Figure 1; Scale bars are 20 µm. *D)* Western blots of total retina lysates probed with anti-HCN1, with PDC used as a loading control. *E)* Relative amount of HCN1 expressed in the retina of wild type, HCN1 heterozygous, and TRIP8b^−/−^ mice as measured using semi-quantitative Western blots.

Reciprocally, TRIP8b can control the subcellular localization of HCN1, particularly in neurons where HCN1 is restricted to a subset of dendrites [24]. However, HCN1 does not display this restricted expression pattern in the retina, so we tested if there is any alteration in HCN1 due to the loss of TRIP8b. In the TRIP8b 1b/2^−/−^ retina, which lacks 5 of the 7 TRIP8b isoforms [26], [29], [31], but still expresses 2 of the 3 isoforms present in photoreceptors (1a and 1a-4), immunolabeling with anti-HCN1 antibodies revealed no change in the compartmentalization of HCN1 (compare Figure 5A and B). In the total TRIP8b^−/−^ retina, HCN1 labeling was reduced in signal intensity but present in the same compartments as in wild type retinas (compare Figure 5A and C). The signal intensity of HCN1 in the IPL was least affected by the loss of all TRIP8b isoforms. Western blotting demonstrated that the levels of total HCN1 protein was not changed in the TRIP8b 1b/2^−/−^ retina but reduced by ∼40% in TRIP8b^−/−^ retinas (Figure 5D). Interestingly, the reduction in HCN1 levels due to either haploinsufficiency of HCN1 or loss of TRIP8b is indistinguishable (Figure 5E).

**Figure 6:**
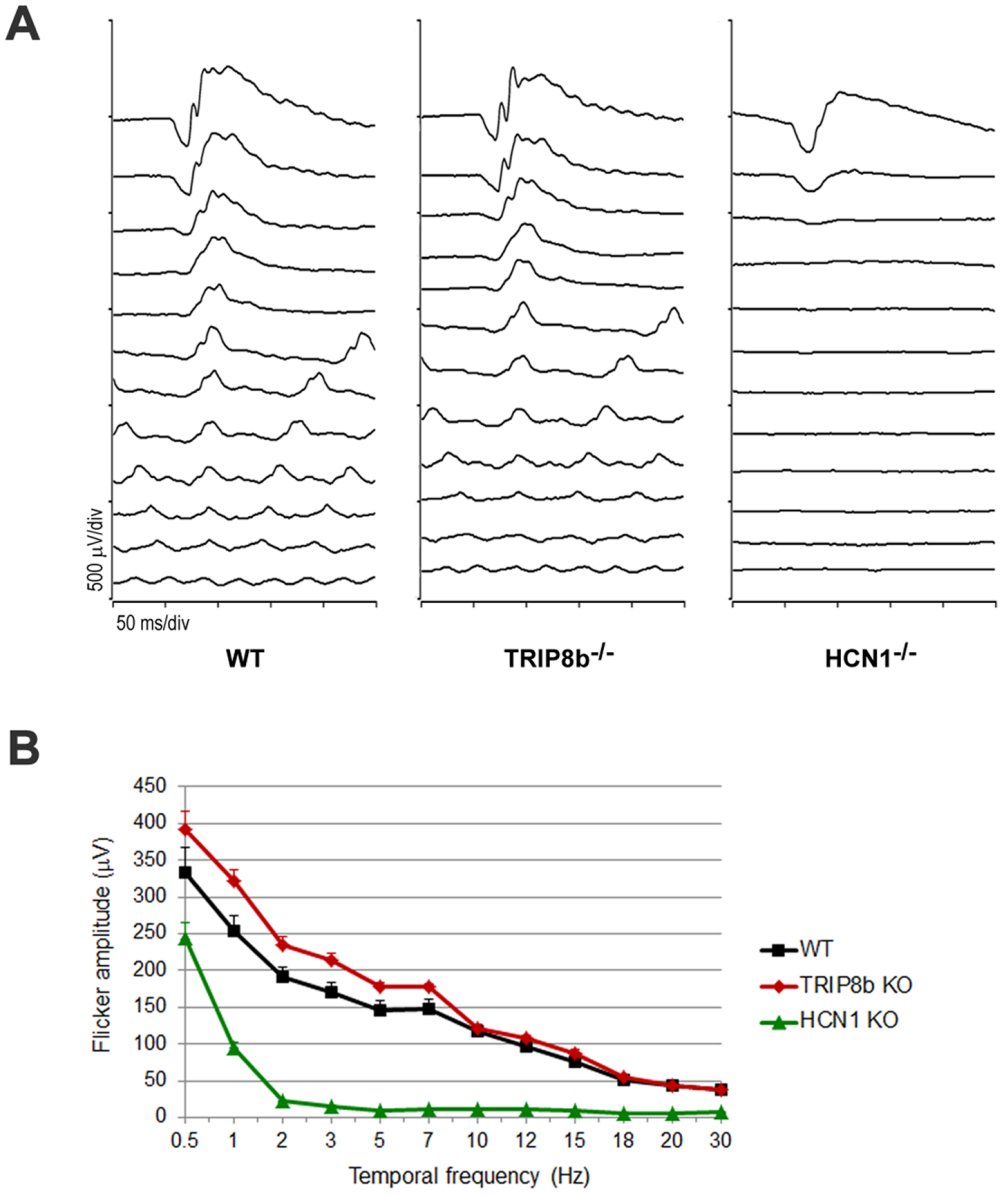
Flicker frequency responses are normal in the absence of TRIP8b. *A)* Representative waveforms from flicker ERGs of WT, TRIP8b^−/−^, and HCN1^−/−^ (n = 3 mice for each genotype). Dark-adapted mice were stimulated with a flash intensity of 3.1 cd.s/m^2^ at frequencies of 0.5, 1, 2, 3, 5, 7, 10, 12, 15, 18, 20, and 30 Hz. *B)* ERG amplitudes plotted as a function of flicker frequency.

To determine if the reduced levels of HCN1 in the retinas of TRIP8b^−/−^ animals compromise retina function we recorded electroretinograms (ERG). One of the major phenotypes in ERG responses from HCN1^−/−^ animals is the failure to resolve high frequency flickering light stimuli; this suppression of cone responses is hypothesized to be driven by abnormally prolonged signaling in rods [12], [14], [40], [41]. Similar defects in ERG responses are expected in TRIP8b^−/−^ mice if the TRIP8b-dependent 40% decrease in HCN1 affects vision. Responses to a single flash under dark or light adapted conditions were similar between TRIP8b^−/−^ and wild type animals (data not shown). In response to a dark-adapted flicker frequency series, HCN1^−/−^ mice failed to respond to cone-isolating frequencies as expected, but responses from TRIP8b^−/−^ animals were not significantly different from that of wild type (p>0.2 by ANOVA; Figure 6). This demonstrates that the decreased HCN1 levels in TRIP8b^−/−^ animals are sufficient for ERG responses.

### HCN1 Surface Expression in the Absence of TRIP8b

**Figure 7:**
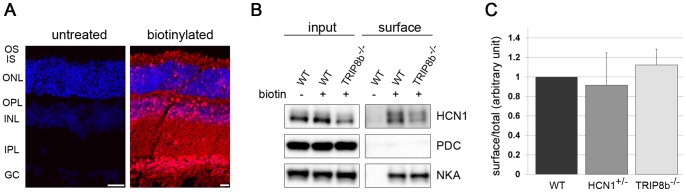
The surface expression of HCN1 is maintained in TRIP8b^−/−^. *A)* Streptavidin staining of control (left) and biotinylated retina (right). *B)* After biotinylation, surface proteins from either control wild type (WT) or TRIP8b^−/−^ mice were pulled down using NeutrAvidin beads. The level of HCN1 in the total (input) and surface (eluted from NeutrAvidin beads) pools was detected by Western blotting. PDC and NKA were used as negative and positive controls, respectively. *C)* Densitometry of Western blots represented in (B) was used to calculate the surface to total ratio of HCN1 after normalization to the loading control NKA.

TRIP8b can regulate the trafficking of HCN1 to the plasma membrane. To investigate HCN1 surface levels in retinal neurons we used an *ex vivo* biotinylation assay. Freshly isolated retinas were incubated with a non-membrane permeable biotin reagent to cross-link surface proteins to biotin. Labeling sectioned retinas with fluorescently conjugated streptavidin verified that the biotinylation reagent penetrated all regions of the retina (Figure 7A). After labeling with biotin, surface proteins were isolated using NeutrAvidin-agarose beads and analyzed by Western blotting. The labeling of NKA was monitored as a positive labeling control and PDC as a negative labeling control to confirm that only membrane proteins were biotinylated in the assay. HCN1 was readily detected on the surface of retina neurons using this approach and we observed a decreased amount of biotinylated HCN1 in the TRIP8b^−/−^ retinas (Figure 7B). To take into account the different abundance of HCN1 in these two mice we compared the ratio of surface to total HCN1 and found that it was unchanged in the absence of TRIP8b. This ratio is similarly preserved in HCN^+/−^ animals which have a TRIP8b independent reduction in HCN1 (Figure 7C).

## Discussion

In this study we characterized the expression of TRIP8b in the retina. TRIP8b is an accessory subunit of HCN channels that can modulate the function and location of HCN channels in various neuronal populations. We found that TRIP8b is expressed in multiple neurons of the retina and colocalizes with HCN1 in photoreceptors and in the inner plexiform layer. The interaction between these two proteins was confirmed by co-immunoprecipitation and supported by the dependence on HCN1 for membrane recruitment of TRIP8b. TRIP8b undergoes alternative splicing, generating distinct isoforms that can regulate HCN channels in opposing ways. All the major splice isoforms are expressed in the retina and three different forms were found in photoreceptors alone (1a, 1a-4, 1a-2-4). TRIP8b 1a was reported to inhibit the axonal localization of HCN1, and may up- or down-regulate HCN1 surface expression depending on the cell type in which it is expressed. Isoforms 1a-4 has also been shown to regulate HCN1 compartmentalization [29], and both 1a-4 and 1a-2-4 can up-regulate HCN1 surface expression [29], [31], [39].

This observation led us to test three predictions arising from the hypothesis that TRIP8b regulates trafficking of HCN1– HCN1 expression levels, compartmentalization, or surface expression should be altered with the loss of TRIP8b. We used two different lines of TRIP8b knockout mice, TRIP8b 1b/2^−/−^ which lacks just the minor splice isoforms (retaining expression of the more abundant 1a and 1a-4 isoforms), and TRIP8b^−/−^ lacking all isoforms. We did not observe any changes in HCN1 so long as the 1a and 1a-4 isoforms were intact. In the TRIP8b^−/−^ retina, the most striking observation was a 40% down regulation of HCN1 protein levels. Similar TRIP8b dependent decreases in HCN1 have been observed in other neuronal populations and this is likely due to excessive routing of HCN1 to lysosomes in the absence of TRIP8b [26]. The staining intensity of HCN1 in the inner plexiform layers was similar in wild type and TRIP8b^−/−^ mice but notably decreased in the photoreceptor layers. This indicates that TRIP8b has the largest influence on HCN1 in photoreceptors.

Vertebrate photoreceptors are composed of 4 major subcellular compartments: outer segment, housing the phototransduction cascade; inner segment, responsible for many housekeeping functions; nuclear layer; and synaptic terminal, for communication to second order neurons. HCN1 is most abundant in the inner segments and found to a lesser degree in the synaptic layers. Since the TRIP8b 1a isoform can inhibit axonal localization in both hippocampal CA1 pyramidal and medial perforant path neurons [29], [31], we considered that it might also limit the amount of HCN1 that accumulates in the photoreceptor synaptic terminal. This is not the case as the compartmentalization of HCN1 was unaltered with the loss of all TRIP8b isoforms. We also tested if trafficking to the plasma membrane of retinal neurons was altered in the absence of TRIP8b using surface biotinylation assays. Interestingly, the ratio of surface to total HCN1 in retinal neurons was maintained even with the reduction of available HCN1. This indicates that the trafficking of HCN1 in retina neurons occurs independently of TRIP8b.

Our findings differ from the situation in hippocampal CA1 and cortical layer V pyramidal neurons in which TRIP8b is required for the trafficking of HCN1 in distal dendrites [29]. This difference is not completely unexpected as in the retina HCN1 is most abundantly expressed in photoreceptors which lack dendrites. The outer segment is the subcellular compartment functionally analogous to a dendrite and HCN1 is excluded from this compartment. Interestingly, a recent study has demonstrated that TRIP8b is not needed for the pre-synaptic localization of HCN1 in entorhinal cortex [32]. Our results further extend the concept that the trafficking of HCN1 outside of dendrites can be maintained independently of TRIP8b.

It is possible that TRIP8b has a larger role in controlling the localization of HCN1 than we have been able to observe here. For instance, if the specific TRIP8b isoforms expressed in each cell type play opposing roles then deleting or overexpressing the individual isoforms may lead to a more striking phenotype. Or, it is possible that another binding partner of HCN1 compensates for the loss of all TRIP8b isoforms. Two intriguing candidates are caveolin and filamin A, which can both promote clustering of HCN channels on the cell surface [42], [43]. Caveolin is a major component of caveolae which are specialized plasma membrane microdomains, and filamin A is an actin binding protein that can serve as an adaptor between plasma membrane proteins, the cytoskeleton and various signaling molecules [44], [45]. Other binding partners for HCN1 include protocadherin-15 (Pcdh15) and KCNE2. HCN1 interacts with Pcdh15 in cochlear hair cell stereocilia, but in photoreceptors they are found in different subcellular compartments [46], [47]. KCNE2 is an accessory subunit for several voltage gated potassium channels including HCN1 and HCN2. It can influence the expression and biophysical properties of HCN1, but has not been investigated in retina [48]–[51]. Characterization of the HCN1– interactome in retina is needed to further our understanding of HCN1 regulation and trafficking.

In the visual system, HCN1 is activated by light induced hyperpolarization of photoreceptors and carries an inward current that is thought to be required to prevent prolonged rod signaling. Consequently, patients taking ivabradine, an HCN channel blocker used to control heart rate, occasionally report the occurrence of phosphenes as a side effect [52], [53]. Despite the striking decease in the amount of HCN1 in the absence of TRIP8b, we did not see a loss of retinal function as measured with ERGs. While the ERG provides a rapid assessment of the summed output of the retina, this approach lacks sensitivity. An in depth electrophysiological study of individual TRIP8b^−/−^ retina neurons would be helpful to determine how this HCN accessory subunit influences the biophysical properties of retina HCN1 and contributes to visual signaling.

HCN channels play multiple roles in the nervous and cardiac systems and their dysregulation can lead to epilepsy, chronic pain, and heart disease [8], [25], [54]. Pharmacological manipulation of HCN expression and function therefore holds promise for the management of numerous diseases. But to effectively target specific diseases it is important to continue dissecting the modes of regulation that ensure the trafficking and function of HCN channels is differentially regulated in various subcellular compartments, cells, and tissues.

## Supporting Information

Figure S1 **Validation of custom anti-HCN1 antibodies.** *A)* Both rabbit anti-HCN1 and a commercially available mouse anti-HCN1 (NeuroMab) detect HCN1 at ∼100 kD on Western blots. Absence of this band in HCN1^−/−^ retina confirms its specificity. A fainter specific band at ∼250 kD likely represents aggregated HCN1 molecules. Both antibodies label smaller non-specific bands (*). *B, D)* Immunostaining of wild type mouse retina with rabbit anti-HCN1 labels the same compartments as the mouse anti-HCN1 antibody. *C, E)* Use of either antibody on HCN1^−/−^ retina reveals faint non-specific background labeling. Therefore, wild type and HCN1^−/−^ retinas were always stained in parallel and the labeling in the knockout control was subtracted from that of the wild type.
(TIF)
