# Supplementary figures and images for: TRIP8b Is Required for Maximal Expression of HCN1 in the Mouse Retina
Source: PLoS One. 2014 Jan 7;9(1):e85850. doi: 10.1371/journal.pone.0085850 (PMC3883711; doi:10.1371/journal.pone.0085850)

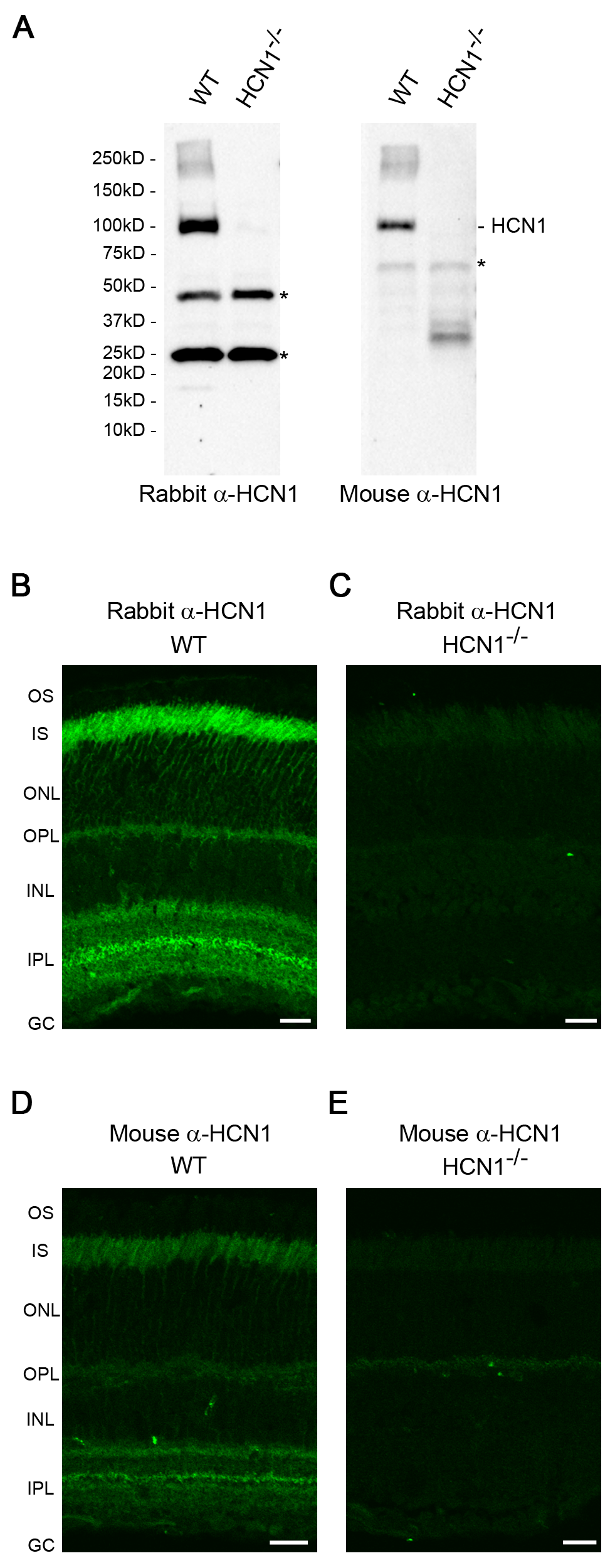

Supplement: Figure S1 — Validation of custom anti-HCN1 antibodies. A) Both rabbit anti-HCN1 and a commercially available mouse anti-HCN1 (NeuroMab) detect HCN1 at ∼100 kD on Western blots. Absence of this band in HCN1−/− retina confirms its specificity. A fainter specific band at ∼250 kD likely represents aggregated HCN1 molecules. Both antibodies label smaller non-specific bands (*). B, D) Immunostaining of wild type mouse retina with rabbit anti-HCN1 labels the same compartments as the mouse anti-HCN1 antibody. C, E) Use of either antibody on HCN1−/− retina reveals faint non-specific background labeling. Therefore, wild type and HCN1−/− retinas were always stained in parallel and the labeling in the knockout control was subtracted from that of the wild type. (TIF) [file pone.0085850.s001.tif]
